# Influence of Vegetable Oils on In Vitro Performance of Lutein-Loaded Lipid Carriers for Skin Delivery: Nanostructured Lipid Carriers vs. Nanoemulsions

**DOI:** 10.3390/pharmaceutics14102160

**Published:** 2022-10-11

**Authors:** Veerawat Teeranachaideekul, Putita Boribalnukul, Boontida Morakul, Varaporn Buraphacheep Junyaprasert

**Affiliations:** Department of Pharmacy, Faculty of Pharmacy, Mahidol University, Bangkok 10400, Thailand

**Keywords:** lutein, virgin coconut oil, rice bran oil, palm oil, nanostructured lipid carriers, NLC, vegetable oils, nanoemulsions, NE

## Abstract

Nanostructured lipid carriers (NLC) were prepared from solid lipid (glyceryl monostearate, GMS) and vegetable oils, including palm oil (PO), rice bran oil (RBO) or virgin coconut oil (VCO), at different ratios (95:5, 90:10 and 80:20), while nanoemulsions (NE) were prepared with sole vegetable oils. After production, the particle size of the lutein-free NLC and NE was found to be between 100 and 150 nm and increased after loading with lutein. An increase in oil loading in NLC reduced the particle size and resulted in a less ordered lipid matrix and an increase in % entrapment efficiency. From the stability study, it was observed that the types of oils and oil content in the lipid matrix had an impact on the chemical stability of lutein. Regarding the release study, lutein-loaded NE showed higher release than lutein-loaded NLC. Both NLC and NE prepared from VCO exhibited higher release than those prepared from PO and RBO, respectively (*p* < 0.05). In contrast, among the formulations of NLC and NE, both lutein-loaded NLC and NE prepared from RBO showed the highest permeation through the human epidermis due to the skin enhancement effect of RBO. Based on all the results, the lipid nanocarriers composed of RBO could effectively enhance the chemical stability of lutein and promote drug penetration into the skin.

## 1. Introduction

Lutein is a member of the xanthophyll family of carotenoids, which contains other carotenoids such as beta-carotene. The xanthophyll family is a unique constituent of a healthy diet and plays an important role in the network of antioxidant vitamins and phytochemicals [1]. Lutein is not produced by the human body and must be consumed from one’s diet. It is abundantly found in eyes and skin, which are two organs of the human body that are susceptible to the potentially deleterious effects of light exposure. It is an antioxidant, anti-stress, anti-inflammatory and blue light filter, which protects the skin from UV damage [2]. The enhancement of skin photoprotective activity of lutein has been reported after oral-and topical administrations [3]. Furthermore, an improvement in skin elasticity (~56%) and hydration (~62%) could be achieved after oral administration for 12 weeks. More improvement was reported when combined with topical lutein (68% for skin elasticity and 82% for skin hydration), which generated even better results than oral or topical lutein individually [3]. According to the literature, the presence of lutein in the skin is important to maintain healthy and proper functions of the skin [3,4,5,6]. As a result, the use of lutein in many cosmetic and pharmaceutical products has received particular attention during the last decade.

Regarding its molecular structure, lutein contains many conjugated double bonds, which can be readily isomerized, oxidized and degraded [7]. Shi and Chen investigated the stability of lutein under different conditions. The results showed that exposure to white fluorescent light at 25 °C accelerated the degradation of lutein at 0.8–10.7% per day, whereas storage in darkness could retard lutein degradation [8]. Moreover, low pH and high storage temperature (e.g., 50 °C) also stimulated lutein degradation [9]. To improve the chemical stability of lutein, numerous techniques have been applied, such as encapsulation in solid lipid nanoparticles (SLN) [10,11], nanostructured lipid carriers (NLC) [10,12], microcapsules [13], nanocapsules [14], liposomes [15] and nanoemulsions (NE) [16].

It was reported that the chemical stability of lutein was improved when encapsulated in poly-ε-caprolactone nanocapsules. The results showed that the percentage of lutein that remained in the nanocapsules was 21% and 33.7% after 90-day storage at 25 °C and 4 °C, respectively, while the half-life of the lutein solution stored at 25 °C was only 10 days [14]. Mitri et al. reported that stability of lutein was enhanced after exposure to 10 MED (minimal erythema dose) of UV radiation when incorporated into lipid nanoparticles, i.e., SLN and NLC, compared to NE and lutein powder suspended in corn oil. In addition, lutein-loaded SLN and NLC prepared from different solid lipids possessed different chemical stability, in vitro release, and pig ear skin permeation [10]. Recently, the optimized SLN formulation of lutein, glyceryl monostearate, lecithin, and poloxamer 188 at the ratio of 1:3.37:1.78:2.58 (*w*/*w*/*w*/*w*) was reported to have greater stability and sustained release effect when compared to free lutein [11]. Given the fact that solid lipids have reportedly affected the stability and release of lipid nanoparticles, the effect of liquid lipids or oils on the in vitro performance of lipid nanocarriers is rarely elucidated.

Vegetable oils are natural products of plant origin, consisting of ester mixtures derived from glycerol with different degrees of unsaturated C14–C20 fatty acids. Many vegetable oils including palm oil (PO), virgin coconut oil (VCO, and rice bran oil (RBO) are used in cosmetic products, owing to their skin benefits, such as skin hydration, antioxidant, and emollient properties [17,18,19]. VCO contains 92% of saturated fatty acids (in the form of triglycerides) and most of them (about 62–70%) are medium-chain fatty acids [20]. RBO contains approximately 80–85% unsaturated fatty acids [21], whereas PO comprises approximately equal amounts of saturated and unsaturated fatty acids [22]. The difference in compositions of these vegetable oils may possess different physicochemical properties and benefits. Therefore, incorporation of the different vegetable oils into NLC may affect the inner structure and performance of NLC for dermal delivery.

In the present work, lutein-loaded NLC (L-NLC) and NE (L-NE) containing PO, VCO, or RBO were developed by hot high-pressure homogenization. The effects of these vegetable oils on the physicochemical properties of L-NLC, including the mean particle size, size distribution (PDI), zeta potential, crystallinity and long-term stability, were observed and compared with L-NE. Moreover, the in vitro performance of L-NLC and L-NE for dermal delivery was evaluated in terms of occlusion factor, release study and skin permeation through human epidermis.

## 2. Materials and Methods

### 2.1. Materials

Lutein oil suspension (20%) in safflower oil was purchased from DSM Nutritional Product Ltd. (Basel, Switzerland). Glyceryl monostearate (GMS) was obtained from Croda (Nettetal, Germany). Poloxamer 188 was acquired from BASF (Ludwigshafen, Germany). RBO was purchased from Thai Edible Oil (Bangkok, Thailand). VCO was from Tropical Nutrition (Bangkok, Thailand). PO was provided by Lam Soon (Bangkok, Thailand). Cetyl palmitate (CP) was obtained from Sabo (Levate, Italy). Trimyristin (Dynasan^®^ 114) was received as a gift from Sasol (Witten, Germany). Tween 80 was purchased from Uniqema (New Jersey, NY, USA). Methanol and acetonitrile were purchased from Burdick & Jackson (Seoul, Korea). Sterile water for injection was acquired from Thai Nakorn Patana (Bangkok, Thailand).

### 2.2. Lipid Screening

In this study, trimyristin, GMS and CP were selected as representatives of triglycerides, monoglyceride and wax, respectively. The suitable solid lipid for preparing NLC was selected based on the miscibility test of lutein in hot melted solid lipid at 80 °C for 1 h [23]. The solid lipid was melted with an accurate amount of lutein at 80 °C using a shaking bath. The dissolved lutein in melted solid lipid was detected by visual observation. The lipid that could dissolve lutein was chosen for NLC preparation.

### 2.3. Preparation of Lutein-Free and Lutein-Loaded Nanoparticles 

In this study, lutein-free NLC (B-NLC) was prepared by the hot high-pressure homogenization (HPH) technique [24,25]. Briefly, the mixtures of lipids (i.e., solid lipid and oil) were melted around 80 °C. The hot aqueous surfactant solution (poloxamer 188 in water) at 85 °C was added to the melted lipid phase under stirring by Ultra-Turrax^®^ T25 (IKA, Staufen, Germany) at 8000 rpm for 1 min. Afterward, the obtained pre-emulsion was homogenized using the APV-2000 high-pressure homogenizer (APV Gaulin, Lübeck, Germany) at 500 bar for 3 cycles. NLC dispersion was obtained after cooling down under ambient conditions to room temperature.

For L-NLC, it was produced in the same manner as B-NLC; however, lutein was added into the melted lipid phase before the addition of hot aqueous surfactant. In addition, lutein-free NE (B-NE) and L-NE were also prepared using the sole liquid lipid as described above. Table 1 shows the compositions of all developed lutein-free and lutein-loaded lipid nanocarriers.

### 2.4. Particle Size Analysis

The mean particle size and the particle size distribution (polydispersity index, PDI) were determined by photon correlation spectroscopy (PCS) using Zetasizer NanoZS (Malvern Instruments, Worcestershire, UK) [26]. The sample was diluted with sterile water for injection to obtain a suitable scattering intensity. The mean particle size and PDI were obtained by averaging the values from 10 measurements at an angle of 173° in a 10 mm diameter cell, at 25 °C. Moreover, laser diffraction (LD) using the Mastersizer 2000 (Malvern Instruments, Worcestershire, UK) was employed to detect the presence of microparticles. The obtained LD data were evaluated using volume distribution as the diameter (d) values of 10%, 50% and 90% and Span value. The diameter values indicate the percentage of particles that possess a diameter equal to or lower than the given value. The Span value is a statistical parameter used to evaluate the particle size distribution and can be calculated by applying the following equation:

(1) $$\mathrm{Span}=\frac{d90\%\;-\;d10\%}{d50\%}\;$$

### 2.5. Zeta Potential Analysis

The zeta potential (ZP), also called the electrokinetic potential, is defined as the value of the electrical potential at the shear plane of particles and indicates the physical stability of colloidal systems. The ZP was measured by Malvern Zetasizer NanoZS by averaging the attained values from 3 measurements at 25 °C. 

### 2.6. Particle Morphology

The particle morphology of NLC was examined using a transmission electron microscope (TEM, Hitachi Model H-7000, Tokyo, Japan). The sample was prepared by the negative staining technique. The sample was dispersed directly into the deionized water and then placed on a copper grid coated with collodion film. Consequently, it was stained with 2% *w*/*v* phosphotungstic acid solution and dried at room temperature. The sample was then investigated at 70 kV.

### 2.7. Differential Scanning Calorimetry (DSC)

The thermal behavior of NLC was evaluated using a differential scanning calorimeter (PerkinElmer^®^ DSC 8000, Waltham, MA, USA). Approximately 1–3 mg of the sample based on its lipid content was accurately weighed into a crimped standard aluminum pan (~20 µL) and sealed with the aluminum pan before analysis. An empty pan was used as a reference. The DSC measurement was conducted over a temperature range of 25–90 °C using a heating rate of 10 °C/min and flushed with nitrogen at a flow rate of 20 mL/min. The melting point, melting enthalpy (ΔH) and onset temperature were recorded. The percentage of the crystallization index (%CI) was calculated by the following equation.

(2) $$\mathrm{CI}\;(\%)=\frac{\;\Delta {H\;}_{\mathrm{aqueous}\;\mathrm{NLC}\;\mathrm{or}\;\mathrm{SLN}\;\mathrm{dispersion}}}{\Delta {H\;}_{\mathrm{bulk}\;\mathrm{material}}{\;\times \;\mathrm{Concentration}}_{\;\mathrm{lipid}\;\mathrm{phase}}}\times 100\;$$

### 2.8. The Percentage of Entrapment Efficiency

The entrapment efficiency of lutein-loaded NLC and NE was determined by the indirect method. The sample was placed in an ultracentrifuge tube and centrifuged at 6000 rpm for 30 min. The free drug in the supernatant was analyzed by the HPLC method. The total lutein concentration was determined by HPLC spectroscopy using a UV detector, regardless of stereoisomers, because all of them are potent antioxidants and also reduce oxidative damage indirectly by light [27]. Briefly, the HPLC system consisted of a 20AD series HPLC machine (Shimadzu, Kyoto, Japan), equipped with a photodiode array detector. The stationary phase was a C18 column with the particle size of 5 μm (Thermo^®^ Hypersil ODS C18, 250 mm × 4.6 mm, Waltham, MA, USA). The mobile phase was composed of methanol and acetonitrile (90:10 *v*/*v*). The flow rate of the mobile phase was 1 mL/min and the eluted sample was detected at 450 nm. The system suitability was determined before analysis. The amount of encapsulated drug was determined as a result of the total amount of drug minus that of the free drug. The percentage of entrapment efficiency (%E.E.) was calculated by the following equation.

(3) $$\%E.E.=\frac{\mathrm{Total}\;\mathrm{amount}\;\mathrm{of}\;\mathrm{drug}\;-\;\mathrm{Amount}\;\mathrm{of}\;\mathrm{free}\;\mathrm{drug}}{\mathrm{Total}\;\mathrm{amount}\;\mathrm{of}\;\mathrm{drug}}\;\times \;100\;$$

### 2.9. Long-Term Stability 

To evaluate the long-term physical and chemical stability of lutein-loaded NLC and NE, the samples were stored at 4 °C and 30 °C and protected from light by being kept in dark conditions for 3 months. The physical stability of lutein-loaded NLC and NE stored at different conditions was studied regarding particle size, size distribution, ZP and %CI. The percentages of lutein remaining and entrapment efficiency after 3 months of storage were determined by the HPLC method, as previously described.

### 2.10. Skin Performance Studies 

#### 2.10.1. Occlusive Effect

The occlusion factor was measured using the in vitro assay as described by de Vringer [26]. In brief, a 50 mL beaker was filled with 20 mL of water and sealed with a 90 mm diameter cellulose acetate membrane (Whatman number 5, cutoff size of 2.5 µm, Buckinghamshire, UK). An exact amount of 400 µL of the sample was homogenously spread over the membrane. Then, the beaker was stored at 32 °C and 75% RH for 24 h. The experiment was carried out in triplicate and the occlusion factor (F) was calculated by the following equation:

(4) $$F=\frac{(A\;-\;B)}{A}\;\times \;100\;$$

where A is the water loss without the sample (reference) and B is the water loss with the sample.

#### 2.10.2. In Vitro Release Study

The in vitro release study was performed using vertical Franz diffusion cells (volume 10 mL, diameter 1.5 cm, Crown scientific, Sommerville, MA, USA). A mixed cellulose esters (MF-Millipore, Cork, Ireland) membrane, with a pore size of 0.05 µm, was mounted between the donor and receptor phase. The receptor medium was 2% *w*/*v* of Tween 80 in phosphate buffer saline (pH 5.5). The temperature of diffusion cells was controlled using a water jacket at 32 °C. The receptor medium was stirred by a magnetic stirrer at 700 rpm. Three hundred microliters of the sample were dropped into the donor phase. At certain time intervals of 1, 2, 4, 6, 8, 10 and 24 h, 500 µL of receptor medium was withdrawn and immediately replaced with the same volume of freshly receptor medium. The samples were analyzed by HPLC method, as previously described. The experiment was carried out in triplicate.

#### 2.10.3. Skin Permeation Study

##### Skin Preparation

The skin permeation study was performed using excised human skin from female patients, who had abdominal plastic surgery at Yahee Hospital, aged between 25 and 60 years. The procedure was approved by the Committee on Human Rights Related to Human Experimentation, Mahidol University, Bangkok, Thailand (MU-DT/PY-IRB 2013/031.0307). After excision, the skin was cut into 10 × 10 cm^2^ per piece, and the subcutaneous fatty tissue was removed from the skin specimen using a scalpel. Consequently, the epidermis was separated from the dermis using the heat separation technique [28]. Briefly, the skin was immersed in hot water at 60 °C for 60–90 s. Afterward, the epidermis was separated from the dermis using forceps. The obtained epidermis was cut into approximately 2 × 2 cm^2^, wrapped with aluminum foil, and stored at −20 °C until use. 

##### In Vitro Skin Permeation Study

The in vitro skin permeation study was performed using vertical Franz diffusion cells (volume 10 mL, diameter 1.5 cm, Crown Scientific, Sommerville, MA, USA). Prior to the experiment, the skin was thawed and hydrated in isotonic phosphate buffer (pH 7.4) overnight in the refrigerator. The skin integrity was observed at the beginning and the end of permeation experiment by determining the electrical resistance of the skin. An electrical system was constructed by connecting a 1.5 V battery in series with a high resistance fixed resistor (100 kΩ) and skin. The electrodes were Ag/AgCl. The voltage across the fixed resistor was measured by a voltmeter and subsequently, the skin resistance was calculated. The receptor medium consisted of a phosphate buffer (pH 7.4) that contained 2% *w*/*v* of Tween 80. The temperature of diffusion cells was set at 37 °C using a water jacket. The human epidermis was placed between donor and receptor phases and equilibrated for 30 min. Then, 300 µL of the dispersion was added to the donor compartment of each Franz diffusion cell. The 500 µL of receptor medium was collected at 1, 2, 4, 6, 8, 10 and 24 h and an equal volume of fresh receptor phase was replenished immediately after sampling. The amount of lutein was analyzed by HPLC method, as aforementioned. 

### 2.11. Statistical Analysis

Statistical analysis of the difference in the physicochemical properties among the predetermined intervals of the same formulation and between formulations was performed by using paired t-tests and one-way analysis of variance (ANOVA), respectively. The level of significance was taken at a *p*-value of 0.05.

## 3. Results

### 3.1. Lipid Screening

In this study, the miscibility of 20% *w*/*w* lutein in safflower oil was analyzed in different solid lipids. The results showed that 1% *w*/*w* of lutein (related to 0.2% *w*/*w* lutein active) could not be dissolved in all selected solid lipids. Therefore, the percentage of lutein in safflower oil was adjusted to 0.5% *w*/*w* (related to 0.1% *w*/*w* lutein active). It was found that lutein showed high solubility and homogeneity with GMS in both molten and solidified states. On the other hand, phase separation was observed for trimyristin and CP after being solidified at room temperature. Therefore, GMS was chosen as a solid lipid for the preparation of NLC.

### 3.2. Particle Size snd Size Distribution of Lutein-Free and Lutein-Loaded Nanoparticles

**Figure 1:**
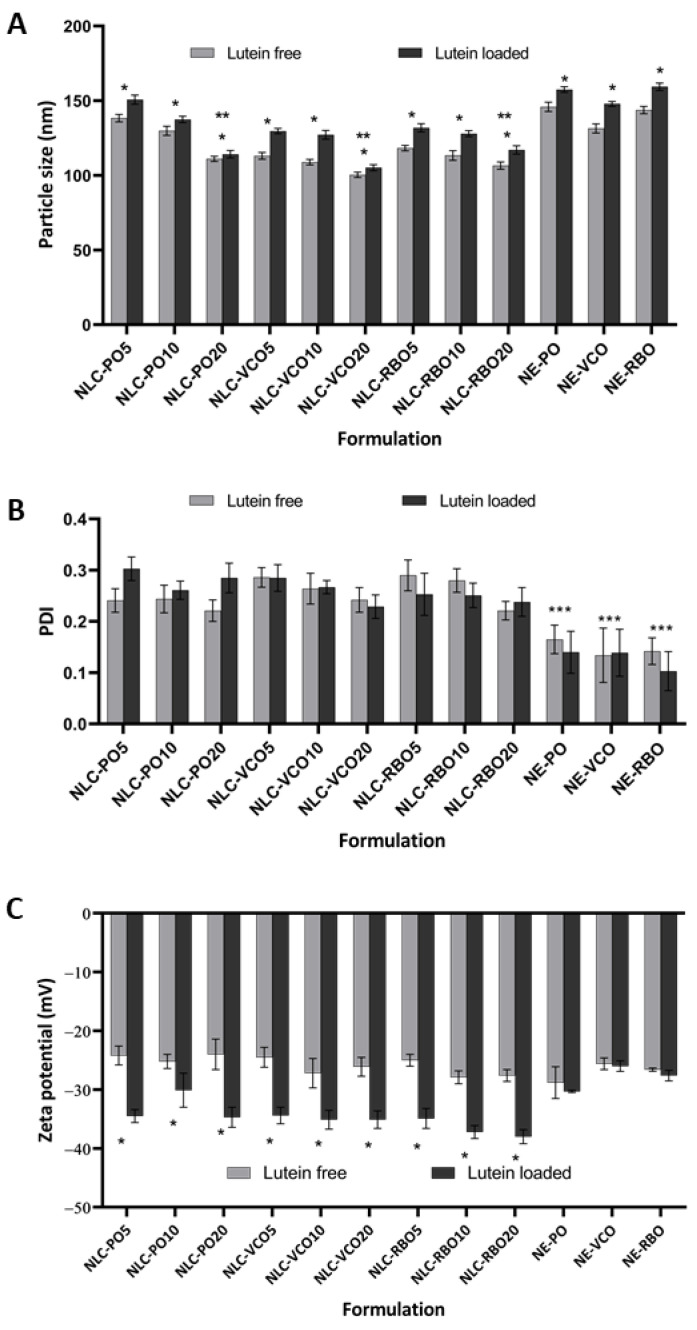
Particle size (**A**), PDI (**B**) and ZP (**C**) of lutein-free and lutein-loaded lipid nanocarriers after production. * Statistically significant difference comparing lutein-free and lutein-loaded nanocarriers (*p* < 0.05), ** statistically significant difference comparing all L-NLC formulations that contain the same type of oil (*p* < 0.05); *** statistically significant difference comparing all lutein-loaded nanocarriers (*p* < 0.05).

To investigate the effect of different vegetable oils on the characteristics of NLC, B-NLC and L-NLC were prepared from three different vegetable oils, including PO, VCO, and RBO, with different ratios of oil loading, as shown in Table 1. In this study, the total concentration of the lipid phase was fixed at 10% *w*/*w*. The ratios of GMS and vegetable oils were varied from 95:5 to 90:10 and to 80:20 for the preparation of NLC formulations. After production, the mean particle size of all B-NLC and B-NE determined by PCS was in the range of 100–150 nm with the PDI lower than 0.35, as shown in Figure 1A,B.

After incorporation of lutein into the nanocarriers, a significant increase in particle size of all developed L-NLC and L-NE was observed (*p* < 0.05), suggesting that lutein was incorporated in the nanocarriers. However, the mean diameters of all developed formulations remained in the nanometer range (105–160 nm) with the PDI lower than 0.35, as shown in Figure 1A,B.

Based on the mean particle size of the NLC system, the particle size of B-NLC and L-NLC tended to decrease with increasing oil loading. As observed in Figure 1A, the 20% oil loading of all vegetable oils provided the lowest particle size, as compared to 10% and 5% oil loading, respectively. This indicated that an increase in oil ratio significantly decreased the particle size (*p* < 0.05), when compared between the formulations that contain the same type of oil. It was due to the fact that the addition of oil into the particle matrix reduced the viscosity, thus easing dispersion and leading to the smaller particles [10,29]. In addition, the particle size of NLC and NE with and without lutein loading also depended on the types of vegetable oils. NLC and NE prepared from VCO showed the lowest particle size compared to those prepared from RBO and PO, respectively, which may be due to the low viscosity of VCO. The viscosity of VCO, RBO and PO at 80 °C was 10.00, 11.49, and 14.16 mPas, respectively. The obtained results suggested that the particle size of NLC and NE prepared from different vegetable oils was affected by the viscosity of oils. Figure 1B depicts the PDI values of NLC and NE with and without lutein loading. It was found that PDI values of less than 0.35 were detected, indicating narrow size distribution. 

Due to the limitation of PCS, which can measure the particles from a few nm to 10 µm, LD was performed to detect the presence of microparticles. Table 2 shows the obtained volume distribution diameter of 10%, 50% and 90% particles of lutein-free and lutein-loaded lipid nanocarriers, respectively. The LD data demonstrated that d50% of all formulations was smaller than 200 nm and the Span values were less than 1. According to the values of d90%, no particle larger than 1 µm was observed, suggesting that no microparticles were detected in any of the formulations. 

The ZP values of B-NLC and B-NE ranged from −24 mV to −29 mV (Figure 1C). The negative charge was likely to be due to the ionized part of fatty acids of GMS and the oils [30]. After incorporation of lutein into NLC and NE, the ZP values of all formulations became more negative. The ZP of L-NLC and L-NE was about −26 mV to −38 mV, respectively, as shown in Figure 1C, implying that all lutein-loaded formulations were physically stable. 

### 3.3. Particle Morphology

**Figure 2:**
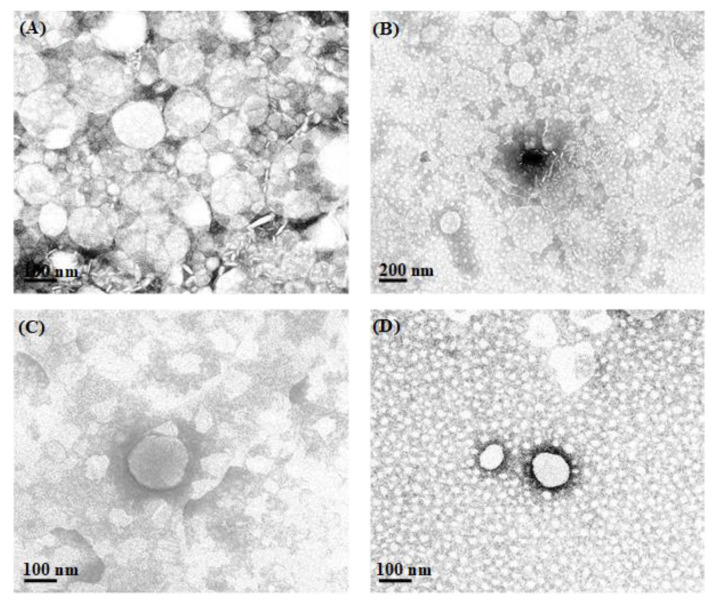
TEM images of lutein-loaded lipid nanocarriers of L-NLC-RBO5 (**A**), L-NLC-RBO10 (**B**), L-NLC-RBO20 (**C**) and L-NLC-VCO20 (**D**).

Figure 2 shows examples of the TEM images of L-NLC after being stained with 2% *w*/*v* phosphotungstic acid solution. The morphological features of L-NLC showed spherical particles less than 200 nm in size. The data obtained from TEM were well correlated with those from PCS.

### 3.4. Differential Scanning Calorimetry (DSC)

**Figure 3:**
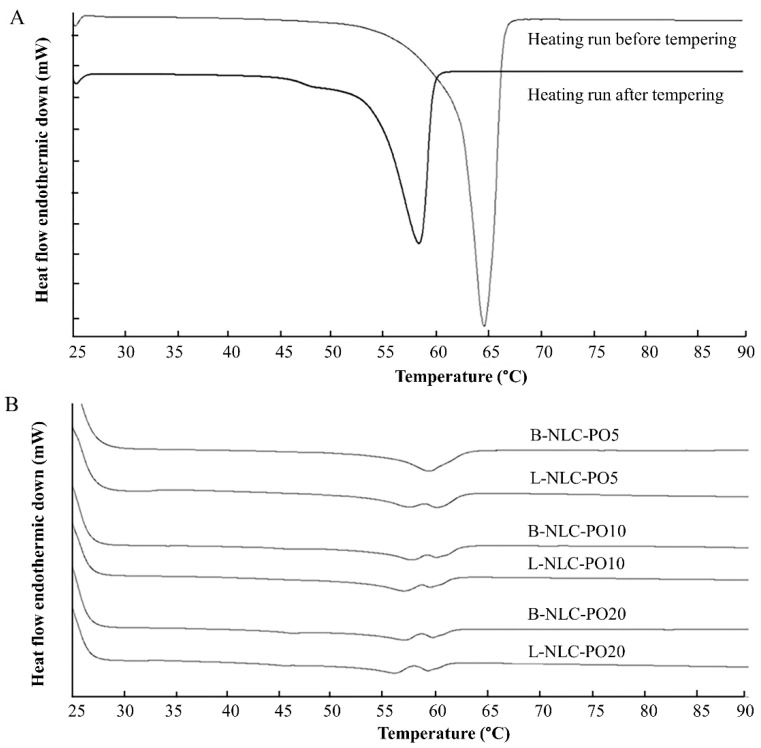
DSC thermograms of GMS before and after tempering at 90 °C for 15 min (**A**); a B-NLC and L-NLC containing 5, 10 and 20% of PO loading (**B**).

The DSC measurement is widely used to evaluate thermal behaviors of lipids in lipid nanoparticles. After the preparation of nanoparticles, the lipid modification is distinguished by DSC on the basis of changes in melting points and enthalpies [31]. In this study, the solid lipid (GMS) and the physical mixtures of GMS and different vegetable oils at various ratios were tempered at 90 °C for 15 min to mimic the production condition (Table 3). The melting point of GMS was revealed at 64.67 °C with an onset of 62.11 °C, corresponding to β modification [32]. After tempering, the melting peak and onset temperature of GMS decreased to 58.14 °C and 54.06 °C, respectively, as shown in Figure 3A. The decrease in onset temperature and the peak of bulk GMS were due to the polymorphic transition from the β or β’ modification to the α modification [33]. This implied that the production temperature influenced the polymorphism of GMS. 

Regarding the effect of vegetable oil on the thermal behavior of GMS (Table 3), it was found that the increasing vegetable oil content decreased the onset, melting peak and %CI of GMS. This indicates either the interference of the oil on the crystallization of the solid lipid or the presence of lattice defects (less ordered structure).

Regarding the DSC thermograms of L-NLC that contained 5% vegetable oil, a melting peak pattern similar to the bulk material (Figure 3) was observed. In contrast, the addition of 10–20% vegetable oil and/or incorporation of lutein caused a distinct change in melting behaviors. Figure 3B shows examples of DSC thermograms of B-NLC and L-NLC prepared from PO. The changed melting behaviors might be due to the presence of an imperfect crystal lattice, owing to the high loading of the vegetable oil in the GMS nanoparticles. In general, nanoparticles produced from mixed triacylglycerols are generally found to show more imperfections in the crystal lattice, while the ones made from monoacid triacylglycerols have a higher degree of crystalline order [34]. From Table 3, it can be observed that the %CI decreased with the increasing amount of oil, meaning that the less ordered crystal was more pronounced. 

### 3.5. The Percentage of Entrapment Efficiency (%E.E.)

The qualitative analysis of lutein was investigated by HPLC spectroscopy using a UV detector. The retention time of lutein was 4.7 min. The calibration curve of lutein was performed in the concentration range of 0.1–25.0 μg/mL. The coefficient of determination (r^2^) was higher than 0.999. The relative standard deviation of intraday and interday precision was less than 2.0%. The recovery was in the range of 95–105%. The limit of quantification (LOQ) and limit of detection (LOD) were 0.09 and 0.03 μg/mL, respectively. 

The %E.E. of L-NLC and L-NE was assayed by HPLC and calculated based on Equation (3). Table 4 shows the %E.E. of all freshly developed formulations. It was found that the %E.E. of all formulations was in the range of 94.0–99.5%.

After comparing the effect of oil loading on the %E.E., it was observed that L-NLC containing 20% oil showed significantly higher %E.E. than those that consisted of 5% oil (*p* < 0.05). It indicates that the drug entrapment efficiencies increased with increasing the amount of oil loading. The incorporation of liquid lipids could disturb the large crystal order of solid lipids and increase the degree of imperfections in the lipid matrix [35,36]. Thus, it provided more space to accommodate active ingredient molecules and led to an improved drug payload. However, %E.E. of L-NLC at the same concentration of different oils was comparable (*p* > 0.05). Therefore, the type of vegetable oils did not affect the %E.E. of L-NLC.

### 3.6. Long-Term Physical and Chemical Stability of L-NLC and L-NE

#### 3.6.1. Physical Stability

**Figure 4:**
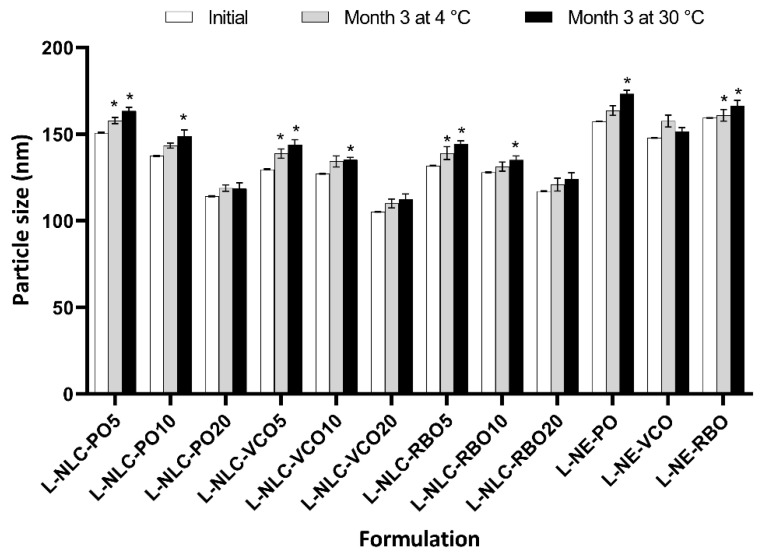
Particle size of lutein-loaded lipid nanocarriers after production (day 0) and after storage for 3 months at 4 °C and 30 °C. * Statistically significant difference comparing day 0 and 3 months of storage (*p* < 0.05).

All samples were stored at 4 °C and 30 °C for 3 months. After 3 months of storage, the color of all formulations at 30 °C changed from dark orange to pale yellow, while no change in color of these formulations stored at 4 °C was observed. The color change was probably related to the degradation of lutein, implying that the storage temperature could affect the chemical stability of lutein in the formulations. The particle size of all formulations on the day of production and 3 months of storage at 4 °C and 30 °C is shown in Figure 4. The particle size of L-NLC containing 5% oil after being stored for 3 months at both temperatures significantly increased compared to those atinitial (*p* < 0.05). This means that the inclusion of oil into lipid nanoparticles at the suitable amount (10% and 20%) could retard the aggregation of nanoparticles, leading to the improvement of the physical stability of NLC. 

Concerning the effect of temperature on the particle size, the results indicate that the suitable storage condition could preserve or prolong the physical stability of lutein-loaded nanocarriers. The physical stability of lutein-loaded nanocarriers stored at 4 °C was better than that stored at 30 °C. However, the particle size of all formulations after 3 months of storage at both temperatures remained lower than 200 nm.

According to the effect of oil loading, it was observed that no statistical difference in the particle size between an initial time of 0 days of storage and after 3 months of storage at both conditions (i.e., 4 °C and 30 °C) was observed for L-NLC containing 20% oil loading (*p* > 0.05). From the above results, it can be concluded that the inclusion of high content of oil into these nanoparticles could retard the aggregation.

Based on the LD data, there was no microparticle presence in all formulations after 3 months of storage under both conditions. The ZP of all formulations remained higher than |−30 mV| after being stored under 4 °C and 30 °C, indicating that all developed lutein-loaded nanocarriers showed good physical stability for at least 3 months at 4 °C and 30 °C.

#### 3.6.2. Chemical Stability

**Figure 5:**
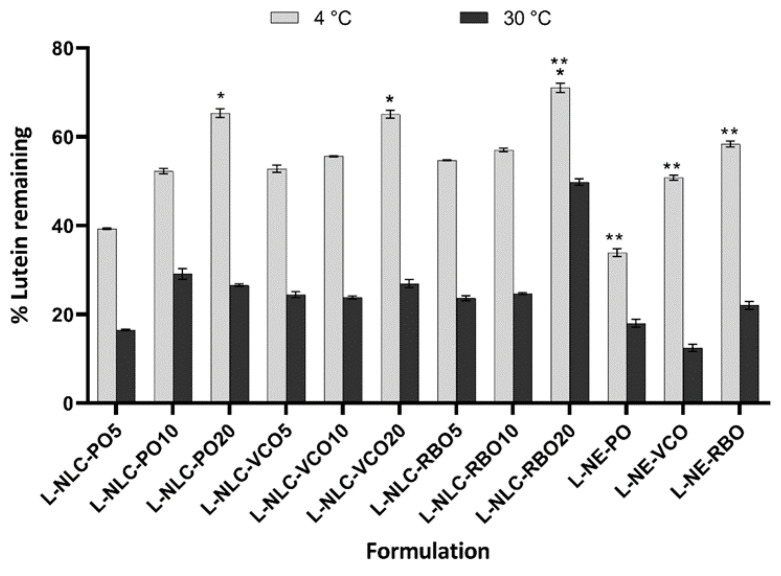
The % lutein remaining after being stored for 3 months at 4 °C and 30 °C (*n* = 3). * Statistically significant difference among all L-NLC formulations that contained the same type of oil (*p* < 0.05). ** Statistically significant difference comparing the % of lutein remaining among all L-NLC and L-NE formulations that contained the same concentration of oil (*p* < 0.05).

The % lutein remaining in all formulations was determined after storage at 4 °C and 30 °C for 3 months, as shown in Figure 5. It was noticeable that the % lutein remaining in all formulations tended to decrease upon storage, depending on the storage temperature. Among all formulations, the % lutein remaining of lutein-loaded nanocarriers stored at 30 °C was significantly lower than those stored at 4 °C (*p* < 0.05). The percentage of lutein remaining after being stored at 30 °C for 3 months was lower than 30%, except for L-NLC prepared from 20% RBO loading (~49.8%).

Concerning the chemical stability of lutein-loaded nanocarriers stored at 4 °C for 3 months, L-NLC containing 20% vegetable oil in the lipid matrix yielded significantly higher % of lutein remaining than that containing 5 and 10% vegetable oil formulations (*p* < 0.05), regardless of the types of vegetable oils. This indicates that the high % of lutein remaining of L-NLC resulted from the high oil loading. It might be due to the inclusion of the oil in the lipid matrix that could retard the formation of the perfect lattice in the lipid matrix, and thus reduced the expulsion of lutein from the lipid matrix. As a result, encapsulation of lutein in the high oil content (e.g., 20%) of the NLC matrix could prevent oxidative degradation, leading to the high % of lutein remaining.

Regarding NE system, L-NE prepared from RBO showed a higher % of lutein remaining than those prepared from VCO and PO (*p* < 0.05). This result was attributed to the different compositions of the oils. It is known that the higher degree of unsaturation of vegetable oils is associated with faster oxidative deterioration. The oxidation leads to the formation of highly reactive species, such as alkyl and peroxyl radicals, which can increase the degradation of lutein [37]. It was reported that the amount of unsaturated fatty acid found in VCO, PO and RBO was approximately 7.5%, 50% and 75%, respectively [38,39,40]. By this consideration, lutein would probably be more stable in VCO due to its lowest degree of unsaturation of oil content. However, the chemical stability is also affected by the presence of endogenous antioxidants, such as tocopherols and tocotrienols. It has been reported that gamma-tocopherol exerted a favorable antioxidative effect for lutein [36]. The tocopherols of 26.2, 20.0 and 18.8 mg/100 g and tocotrienols of 93.6, 0 and 50.5 mg/100 g were reported to be found in RBO, VCO and PO, respectively [38,39,40]. As such, RBO contains higher amounts of tocopherols and tocotrienols than PO and VCO. Therefore, the higher stability of lutein in the formulation that contained RBO was probably due to the presence of the higher contents of tocopherols and tocotrienol in RBO, leading to the slower degradation of lutein. Similar to L-NE, L-NLC prepared from RBO showed the highest stability of lutein as compared to those prepared from PO and VCO, especially L-NLC of 20% RBO at 4 °C (% of lutein remaining >70%). Therefore, selecting the suitable oil for the preparation of lipid nanocarriers could provide the higher chemical stability of lutein.

### 3.7. Skin Performance of Nanocarriers

Based on the %E.E. and stability studies, L-NLC containing 20% oil loading was selected for the further studies. To evaluate the performance of nanocarriers for dermal delivery, the occlusion factor, drug release, and skin permeation studies were examined.

#### 3.7.1. Occlusive Effect

**Figure 6:**
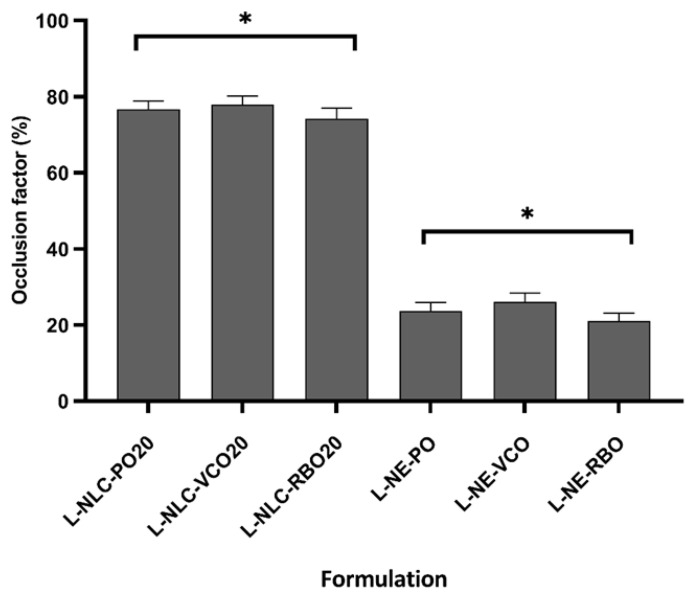
Occlusion factors of L-NLC and L-NE dispersions at 24 h. * Statistically significant difference compared between L-NLC and L-NE (*p* < 0.05). The data are expressed as the mean ± SD (*n* = 3).

The occlusion factor of L-NLC containing 20% oil loading and L-NE is illustrated in Figure 6. The occlusion factor of L-NE was significantly lower than that of L-NLC containing 20% oil loading (*p* < 0.05). This result was explained due to the solid properties of the L-NLC matrix that could prevent the evaporation of water through the filter paper [26].

After comparing the occlusion among L-NLC prepared from different oils, no statistical difference in occlusion factor was observed (*p* > 0.05). This was due to the particle size and %CI of all L-NLC containing 20% oil loading in the same magnitude. As known, the occlusion factor is dependent on the particle size and %CI of lipid nanoparticles [26,41]. When the lipid particle is applied the skin, a hexagonal packing in a monolayer is assumed. Compared to small particles, the holes between large particles are relatively greater in size; therefore, the evaporation of water from larger pore is more pronounced [42]. In addition, the occlusion factor depends on the %CI. It was reported that the higher the %CI, the higher the occlusion factor [41]. Consequently, both parameters should be optimized because the occlusion factor is the major factor to enhance the skin permeation of lipid nanoparticles [43].

#### 3.7.2. Release Study

**Figure 7:**
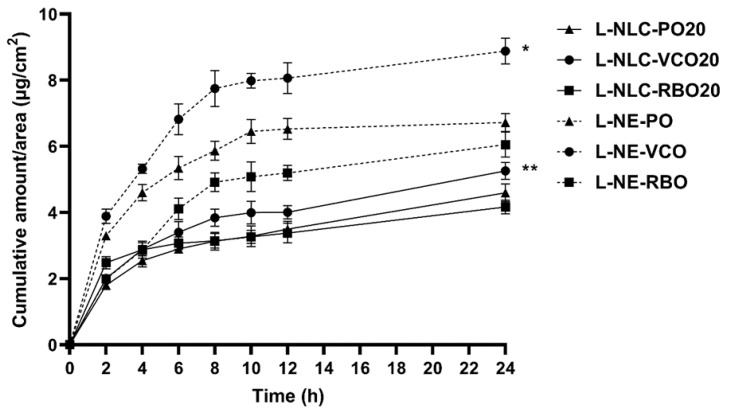
In vitro release profiles of lutein-loaded lipid nanoparticles (*n* = 3). * Statistically significant difference comparing the cumulative amount of lutein release per area at 24 h among all NE formulations (L-NE-VCO, L-NE-PO and L-NE-RBO) (*p* < 0.05). ** Statistically significant difference comparing the cumulative amount of lutein release per area at 24 h among all NLC formulations (L-NLC-VCO20, L-NLC-PO20 and L-NLC-RBO20) (*p* < 0.05).

The in vitro release profile of lutein from 20% oil loading of L-NLC and L-NE was compared, as shown in Figure 7. The amount of lutein released from L-NE was significantly higher than that released from L-NLC. The slow and sustained release property of L-NLC could be due to the crystalline matrix of NLC. The solid matrix of NLC restricted the diffusional mobility of lutein. The slow release of lutein from our experiment is in agreement with the previous study reported by Mitri et al. [10].

Concerning the effect of oils on lutein release, it was found that the higher release of lutein was observed from L-NLC and L-NE prepared from VCO compared to those prepared from PO and RBO (*p* < 0.05). This finding might be caused by the lower viscosity of VCO compared to PO and RBO. As a result, lutein could diffuse from L-NLC and L-NE prepared from VCO more easily than those containing PO and RBO. Moreover, the long polyene chain of lutein might be more compatible with polyunsaturated fatty acids that are enriched in PO and RBO. Therefore, the lutein release from L-NLC and L-NE that contained PO and RBO might be retarded due to their higher amounts of polyunsaturated fatty acids, as mentioned above.

Furthermore, the release profiles of all formulations were determined to fit with zero-order, first-order, and Higuchi’s model and were compared (Table 4). The results showed that the release of lutein from all formulations followed the Higuchi’s model, indicating the square root of the time-dependent process [44]. The Higuchi kinetic release of lipid nanoparticles was generally well-established [26,44,45].

#### 3.7.3. Skin Permeation

Lipid nanoparticles, including NLC and NE, have been intensively used as nanocarriers for improving skin penetration of many drugs. When applying the drug-loaded lipid nanoparticles on the skin, the nanoparticles arrange themselves to form an occlusion film on the skin surface to enhance skin permeation of the drug released from lipid nanoparticles, due to an increase in skin hydration [43]. According to our knowledge, the required dose of lutein for skin benefits has not been evidently reported; however, the high skin permeation of lutein is expected to result in an improvement in skin conditions. In this study, the amount of lutein that permeated through the human epidermis was evaluated over 24 h. The integrity of the skin was evaluated before and after the experiment by measuring the skin resistance. In general, the skin resistance of equal or higher than 15 k·Ω·cm^2^ indicates good skin integrity [46]. In our study, it was found that the skin integrity was almost constant in the range of 36–43 k·Ω·cm^2^, suggesting that the skin barrier function of the epidermis did not change. Based on our experiment, the sink condition was maintained over 24 h because the lutein concentration in the receptor phase was less than 10–30% of the maximum solubility in the receptor medium [47].

**Figure 8:**
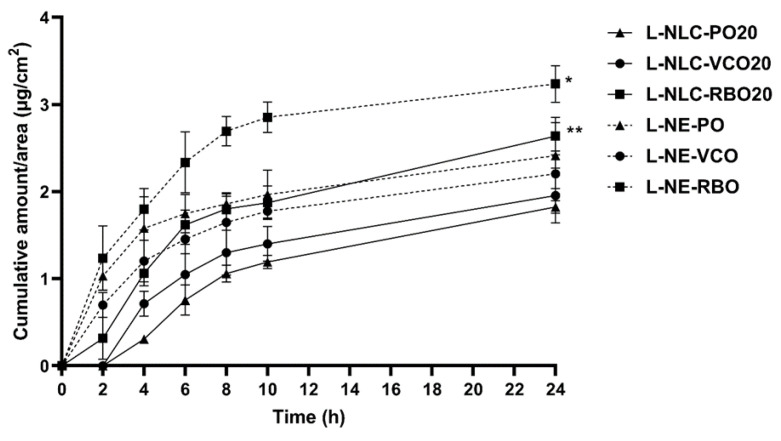
In vitro cumulative amount–time profiles of lutein that permeated through the epidermis from L-NLC and L-NE formulations. * Statistically significant difference comparing all L-NE formulations (*p* < 0.05) and ** statistically significant difference comparing all L-NLC formulations (*p* < 0.05).

Figure 8 illustrates the in vitro skin permeation profiles of L-NLC and L-NE prepared from VCO, PO and RBO for 24 h. After comparing the cumulative amount of lutein in the receptor medium among L-NLC, it was found that L-NLC prepared from RBO showed the highest amount of lutein, followed by those prepared from VCO and PO (*p* < 0.05), respectively. As previously discussed, L-NLC prepared from VCO showed the highest amount of lutein release, followed by L-NLC prepared from PO and RBO, respectively. According to Fick’s law of diffusion, the flux is proportional to the concentration gradient [48]. Therefore, the higher release of L-NLC prepared from VCO was expected to have higher permeation because of the high content of lutein in the donor phase. However, in this study, L-NLC prepared from RBO showed the highest cumulative amount of lutein in the receptor medium, which may be due to the skin enhancement property of RBO. After comparing the compositions of unsaturated fatty acids found in each oil, it can be observed that RBO contains a higher content of unsaturated fatty acids than PO and VCO [46]. Therefore, the high content of unsaturated fatty acids in RBO acts as a skin enhancer to promote the skin permeation of lutein from L-NLC containing RBO. Similar results were also observed for L-NE. The cumulative amount of lutein in the receptor medium obtained from L-NE prepared from RBO was higher than that from L-NE prepared from PO and VCO (*p* < 0.05). Based on the skin permeation study, L-NE and L-NLC that contained RBO could enhance skin permeation, which may subsequently promote the penetration of lutein into the deeper skin layers or dermis.

## 4. Conclusions

Lutein-loaded NLC and NE were successfully produced using the HPH technique with a satisfactory particle size less than 200 nm, a PDI value of lower than 0.350, and a high percentage of encapsulation efficiency. Increasing oil content into the lipid matrix of NLC decreased the particle size and %CI, indicating the presence of the less ordered structure. The different types of vegetable oils did not affect the physical stability (e.g., mean particle size, size distribution, and ZP), but they exerted a significant effect on the chemical stability of lutein. L-NLC and L-NE that contained RBO exhibited higher chemical stability of lutein than those that comprised VCO and PO, owing to the higher content of tocopherol and tocotrienol in RBO. Moreover, the type of vegetable oil also impacted on the release and skin permeation of lutein, due to the difference in the compositions in the oils. As a result, the suitable vegetable oil should be considered for the preparation of lipid nanocarriers for dermal delivery.

## Figures and Tables

**Table 1:** Compositions of lutein-free and lutein-loaded lipid nanocarriers (%*w*/*w*).

Formulation	GMS	PO	VCO	RBO	P188	20% Lutein	Water q.s.
**B-NLC-PO5**	9.5	0.5	-	-	5.0	-	100.0
**B-NLC-PO10**	9.0	1.0	-	-	5.0	-	100.0
**B-NLC-PO20**	8.0	2.0	-	-	5.0	-	100.0
**B-NLC-VCO5**	9.5	-	0.5	-	5.0	-	100.0
**B-NLC-VCO10**	9.0	-	1.0	-	5.0	-	100.0
**B-NLC-VCO20**	8.0	-	2.0	-	5.0	-	100.0
**B-NLC-RBO5**	9.5	-	-	0.5	5.0	-	100.0
**B-NLC-RBO10**	9.0	-	-	1.0	5.0	-	100.0
**B-NLC-RBO20**	8.0	-	-	2.0	5.0	-	100.0
**B-NE-PO**	-	10.0	-	-	5.0	-	100.0
**B-NE-VCO**	-	-	10.0	-	5.0	-	100.0
**B-NE-RBO**	-	-	-	10.0	5.0	-	100.0
**L-NLC-PO5**	9.025	0.475	-	-	5.0	0.5	100.0
**L-NLC-PO10**	8.550	0.950	-	-	5.0	0.5	100.0
**L-NLC-PO20**	7.600	1.900	-	-	5.0	0.5	100.0
**L-NLC-VCO5**	9.025	-	0.475	-	5.0	0.5	100.0
**L-NLC-VCO10**	8.550	-	0.950	-	5.0	0.5	100.0
**L-NLC-VCO20**	7.600	-	1.900	-	5.0	0.5	100.0
**L-NLC-RBO5**	9.025	-	-	0.475	5.0	0.5	100.0
**L-NLC-RBO10**	8.550	-	-	0.950	5.0	0.5	100.0
**L-NLC-RBO20**	7.600	-	-	1.900	5.0	0.5	100.0
**L-NE-PO**	-	9.5	-	-	5.0	0.5	100.0
**L-NE-VCO**	-	-	9.5	-	5.0	0.5	100.0
**L-NE-RBO**	-	-	-	9.5	5.0	0.5	100.0

GMS: glyceryl monostearate, PO: palm oil, VCO: virgin coconut oil, RBO: rice bran oil; P188: poloxamer 188.

**Table 2:** Volume distribution diameters in micrometers (d10%, d50% and d90%) and Span values of freshly prepared lutein-free and lutein-loaded lipid nanocarriers obtained by LD.

Formulations	d10%	d50%	d90%	Span	Formulations	d10%	d50%	d90%	Span
**B-NLC-PO5**	0.132	0.171	0.232	0.587	**L-NLC-PO5**	0.132	0.171	0.233	0.590
**B-NLC-PO10**	0.132	0.171	0.232	0.584	**L-NLC-PO10**	0.132	0.171	0.232	0.586
**B-NLC-PO20**	0.132	0.170	0.231	0.582	**L-NLC-PO20**	0.132	0.170	0.231	0.583
**B-NLC-VCO5**	0.135	0.178	0.244	0.613	**L-NLC-VCO5**	0.138	0.184	0.253	0.626
**B-NLC-VCO10**	0.132	0.170	0.230	0.578	**L-NLC-VCO10**	0.137	0.182	0.250	0.624
**B-NLC-VCO20**	0.132	0.170	0.231	0.581	**L-NLC-VCO20**	0.132	0.170	0.231	0.580
**B-NLC-RBO5**	0.132	0.171	0.232	0.586	**L-NLC-RBO5**	0.132	0.171	0.233	0.589
**B-NLC-RBO10**	0.132	0.171	0.233	0.588	**L-NLC-RBO10**	0.140	0.187	0.258	0.635
**B-NLC-RBO20**	0.132	0.170	0.231	0.584	**L-NLC-RBO20**	0.135	0.187	0.271	0.727
**B-NE-PO**	0.142	0.207	0.250	0.761	**L-NE-PO**	0.145	0.198	0.280	0.682
**B-NE-VCO**	0.135	0.178	0.245	0.614	**L-NE-VCO**	0.133	0.172	0.233	0.583
**B-NE-RBO**	0.142	0.193	0.271	0.669	**L-NE-RBO**	0.138	0.185	0.259	0.652

**Table 3:** The onset, melting endotherm and %CI of physical mixtures of GMS and vegetable oils at different ratios, lutein-free and lutein-load nanocarriers, and bulk GMS after tempering.

Physical Mixtures	Onset (°C)	MP (°C)	CI (%)	Formulations	Onset (°C)	MP (°C)	CI (%)	Formulations	Onset (°C)	MP (°C)	CI (%)
**GMS/PO (95:5)**	52.81	57.74	87.80	**B-NLC-PO5**	54.73	58.98	85.64	**L-NLC-PO5**	56.79	59.73	73.89
**GMS/PO (90:10)**	52.02	57.84	85.13	**B-NLC-PO10**	53.16	57.29	80.47	**L-NLC-PO10**	51.85	56.58	66.46
**GMS/PO (80:20)**	50.83	56.94	72.65	**B-NLC-PO20**	51.56	56.68	60.81	**L-NLC-PO20**	50.86	55.69	50.16
**GMS/VCO (95:5)**	52.77	57.70	92.87	**B-NLC-VCO5**	53.59	58.05	88.32	**L-NLC-VCO5**	51.84	56.25	62.79
**GMS/VCO (90:10)**	51.30	57.44	82.31	**B-NLC-VCO10**	52.58	57.10	85.78	**L-NLC-VCO10**	50.98	55.73	59.21
**GMS/VCO (80:20)**	49.93	56.05	79.12	**B-NLC-VCO20**	51.17	55.84	61.96	**L-NLC-VCO20**	50.47	55.42	47.41
**GMS/RBO (95:5)**	52.90	58.17	91.33	**B-NLC-RBO5**	54.15	58.08	80.95	**L-NLC-RBO5**	51.98	56.25	60.59
**GMS/RBO (90:10)**	51.66	57.44	85.01	**B-NLC-RBO10**	52.52	56.84	78.90	**L-NLC-RBO10**	51.57	55.82	54.87
**GMS/RBO (80:20)**	50.75	56.96	75.04	**B-NLC-RBO20**	52.51	56.97	45.78	**L-NLC-RBO20**	50.06	55.40	41.01
**Bulk GMS (tempering)**	54.06	58.14	100.00								

**Table 4:** The %E.E. of lutein and coefficient of determination (r^2^) of release kinetics of lutein release from L-NLC and L-NE.

Formulations	Entrapment Efficiency (%)	Coefficient of Determination (r^2^)		
		Zero-Order	First-Order	Higuchi’s Model
**L-NLC-PO5**	95.67 ± 1.37			
**L-NLC-PO10**	97.05 ± 1.11			
**L-NLC-PO20**	98.57 ± 2.21	0.8813	0.7494	0.9663
**L-NLC-VCO5**	95.82 ± 1.04			
**L-NLC-VCO10**	97.39 ± 1.05			
**L-NLC-VCO20**	98.71 ± 2.16	0.8203	0.6797	0.9377
**L-NLC-RBO5**	95.67 ± 1.47			
**L-NLC-RBO10**	97.44 ± 2.30			
**L-NLC-RBO20**	98.02 ± 2.36	0.9333	0.8798	0.9743
**L-NE-PO**	99.43 ± 3.05	0.5302	0.4670	0.7368
**L-NE-VCO**	97.57 ± 2.51	0.5857	0.5019	0.7831
**L-NE-RBO**	98.07 ± 1.32	0.6541	0.5358	0.8383

## Data Availability

Data are contained within the article.
